# Oral Resveratrol Prevents Osteoarthritis Progression in C57BL/6J Mice Fed a High-Fat Diet

**DOI:** 10.3390/nu8040233

**Published:** 2016-04-20

**Authors:** Hailun Gu, Keyu Li, Xingyao Li, Xiaolu Yu, Wei Wang, Lifeng Ding, Li Liu

**Affiliations:** 1 Department of Orthopedics, Shengjing Hospital, China Medical University, No. 36 Sanhao Street, Heping District, Shenyang 110004, Liaoning, China; orthowangwei@126.com (W.W.); ding2565@163.com (L.D.); 2 Department of Nutrition and Food Hygiene, School of Public Health, China Medical University, No. 77 Puhe Road, Shenyang North New Area, Shenyang 110122, Liaoning, China; likeyu0715@sohu.com (K.L.); m13804046714@163.com (X.L.); 13322156499@163.com (X.Y.); liuli@mail.cmu.edu.cn (L.L.)

**Keywords:** osteoarthritis, resveratrol, high-fat diet, obesity, apoptosis

## Abstract

The effects of resveratrol on osteoarthritis (OA) pathogenesis have been demonstrated *in vitro* and in animal models employing intra-articular injections. However, the potential for oral resveratrol supplements to mediate protective effects on OA have not been examined. Therefore, the aim of the present study was to investigate the potential anti-OA effects of oral resveratrol on mice fed a high-fat diet (HFD). C57BL/6J male mice were fed either a standard diet or a HFD, and a subset of the latter also received varying doses of resveratrol. Twelve weeks later, all of the animals were sacrificed and knee joints were evaluated with histological, immunohistochemical, and TUNEL analyses. Mice that received a HFD had significantly greater body weights than the control mice and also exhibited features consistent with knee OA. The mice that received a HFD in combination with low, intermediate, or high doses of resveratrol were only slightly heavier than the control mice at the end of 12 weeks. Quantitative histological assessments indicated that resveratrol treatment partly recovered joint structure in the mice that received a HFD, while high doses of resveratrol prevented the degradation of type II collagen into C-telopeptide of type II collagen (CTX-II) and retained type II collagen expression in cartilage. Furthermore, TUNEL analyses revealed a reduction in chondrocyte apoptosis in the resveratrol-treated mice compared with the HFD mice. Thus, oral resveratrol appears to exert anti-OA effects in a mouse model of HFD-induced OA, thereby highlighting the potential preventive and therapeutic value of administering resveratrol for obesity-associated OA.

## 1. Introduction

Osteoarthritis (OA) is a chronic degenerative joint disease that is characterized by the erosion of articular cartilage and osteophyte formation. As a result, individuals with OA experience pain, joint deformation, and even disability. Currently, clinical management of OA focuses on relieving symptoms by reducing pain and inflammation and improving joint function. However, the evolving and pathophysiological processes underlying OA progression are not addressed. Furthermore, long-term use of available pharmacological agents to relieve OA symptoms is associated with substantial gastrointestinal, renal, and cardiovascular side effects [1,2]. Therefore, the development of novel therapies that slow and/or reverse cartilage degradation, and that are also safe to administer for long periods of time, are urgently needed.

Resveratrol is a natural polyphenolic compound that is found in grape skins, berries, and peanuts [3]. *In vitro* studies have demonstrated the capacity for resveratrol to mediate OA-protective effects, including anti-apoptotic, anti-inflammatory, and anti-oxidant effects [4,5,6]. *In vivo* evidence further suggests that resveratrol decreases chondrocyte apoptosis, decreases synovial nitric oxide content [7], and prevents OA progression by activating SIRT1 and silencing HIF-2α [8]. The effects of resveratrol in these studies were observed either *in vitro* or following intra-articular injections of resveratrol directly to the joint, while the potential for resveratrol to mediate OA-protective effects via oral supplementation remains to be investigated.

Animal models of OA have been established in various laboratories to study the mechanisms of OA development [9,10,11,12,13,14,15]. In addition to aging, obesity is one of the most important risk factors for OA [16]. In C57BL/6 mice, obesity induced by a high-fat diet (HFD) has been shown to increase the incidence and degeneration of OA [14,17,18,19]. Obesity precedes the development of OA [20], and thus, is considered to be causally implicated in the degenerative changes that underlie OA [21]. Accordingly, measures to reduce obesity and its related factors are regarded as effective strategies for inhibiting OA progression. Obesity represents a systemic and chronic low-grade inflammation which may be subject to the anti-inflammatory effects of resveratrol. However, the effects of resveratrol on obesity remain controversial. Ikuta and colleagues reported that resveratrol reduced obesity in mice that received a HFD [22], whereas another study demonstrated that only slight changes in body weight were observed following administration of resveratrol [23]. Therefore, the effects and underlying mechanisms of resveratrol on obesity-related OA remain unclear.

The aim of the present study was to investigate the effects of oral resveratrol on OA pathogenesis in C57BL/6J mice that were fed a HFD. To address this objective, we employed a mouse model of knee OA and evaluated its response to a HFD with and without resveratrol. The impact of resveratrol on the expression and degradation product of type II collagen and chondrocyte apoptosis were also examined.

## 2. Materials and Methods

### 2.1. Animals and Treatments

Seven-week-old male C57BL/6J mice were purchased from the Animal Center of China Medical University (Shenyang, Liaoning, China). The animals were randomly divided into five groups of 12 mice to establish a control (CON) group, a HFD group, a low-dose resveratrol (RES-L) group, an intermediate-dose resveratrol (RES-I) group, and a high-dose resveratrol (RES-H) group. Mice in the CON group received a standard diet with 10% of its kilocalories (kcal) derived from fat. Mice in the HFD, RES-L, RES-I, and RES-H groups received a homemade HFD containing 58% of its kcal from fat [24], while mice in the CON and HFD groups received 0.5% carboxymethyl cellulose sodium (CMC, diluted in 0.9% normal saline; Sinopharm Chemical Reagent Co., Ltd., Shenyang, Liaoning, China) by oral gavage. Mice in the RES-L, RES-I, and RES-H groups were also administered 5, 22.5, or 45 mg/kg of resveratrol (Guanyu Biotech, Xi’an, Shanxi, China) diluted in 0.5% CMC by oral gavage, respectively. Food and water were available *ad libitum* and all of the animals received CMC or resveratrol every day for 12 weeks. Body weight and the amount of food intake were recorded each week. Mice were maintained under a constant temperature of 20 ± 2 °C, a relative humidity of 50% ± 10%, and a 12 h light/dark cycle. Animal experimental procedures were conducted according to an animal protocol that was approved by the Institutional Animal Care and Use Committee of China Medical University (Shenyang, Liaoning, China) with a ethic approval code of CMU62033009. Two mice (one in the RES-L group and one in the RES-I group) died during the experimental period.

### 2.2. Serum Triglyceride, Total Cholesterol, and C-Telopeptide of Type II Collagen (CTX-II) Measurements

All mice were sacrificed after 12 weeks. Blood samples were collected from the abdominal aorta and were centrifuged at 1000× *g* for 15 min and stored at −80 °C. Serum triglycerides and total cholesterol were determined using commercial kits (Bio-Technology and Science Inc., Beijing, China). Serum levels of CTX-II were determined by ELISA (Cusabio, Wuhan, Hubei, China). All experimental protocols were performed according to the manufacturers’ instructions.

### 2.3. Assessment of OA

Whole left knee joints were removed by dissection, were fixed in 4% paraformaldehyde for 24 h, and were decalcified in 10% EDTA-Na_2_ for two months at room temperature (RT). After the joints underwent dehydration and paraffin embedding, serial sagittal sections (5 μm) were cut from the whole medial compartment of the joint. The sections were stained with hematoxylin and eosin (H & E) (Tianjin Guangfu Fine Chemical Research Institute, Tianjin, China), Safranin O (Tianjin Guangfu Fine Chemical Research Institute), or Safranin O/Fast Green (Sinopharm Chemical Reagent Co., Ltd., Shanghai, China) and were examined microscopically. All sections were observed and evaluated by two authors (L.K.Y., L.X.Y.). Histological evaluations of cartilage were performed by calculating modified Mankin scores [25] (the original scoring was proposed by Mankin and colleagues [26]). The grading system included four categories: cartilage structure (six points), cartilage cells (three points), staining (four points), and tidemark integrity (two points). A maximum score of 14 points was possible, while normal cartilage received a score of zero points.

### 2.4. Immunohistochemistry

Briefly, tissue sections were incubated overnight at 4 °C with an anti-mouse type II collagen antibody (1:100; Boster Biotechnology, Wuhan, Hubei, China) and then with an appropriate secondary antibody (Boster Biotechnology) at 37 °C. After 30 min, bound antibodies were visualized using peroxidase-conjugated avidin and diaminobenzidine according to the manufacturer’s instructions (Boster Biotechnology). Histomorphometric measurements were made using image analysis software (Image J2X, Media Cybernetics, Carlsbad, CA, USA), and positive cells were defined based on their type II collagen immunostaining. Cartilage areas were selected, and staining signals were quantified and averaged.

### 2.5. TUNEL Assay

TUNEL assays were performed according to the manufacturer’s instructions (Boster Biotechnology, Wuhan, China). Briefly, sections were incubated with proteinase K for 15 min at RT, then were washed with Tris Buffered Saline. The sections were subsequently incubated with a buffer containing TdT and DIG-d-UTP for 2 h at 37 °C in a humidified chamber, were blocked for 30 min at RT, were incubated with a biotin-conjugated anti-digoxin antibody (1:100) for 30 min at 37 °C in a humidified chamber, and were incubated in Strept Avidin-Biotin Complex (SABC) for 30 min at 37 °C. The staining of each section was examined by fluorescence microscopy and the number of TUNEL-positive cells in the cartilage above the tidemark was determined.

### 2.6. Statistical Analysis

Data are expressed as the mean ± standard error (SE). Statistical analysis was performed using one-way analysis of variance (ANOVA; SPSS 13.0 software, SPSS Inc., Chicago, IL, USA). A *p*-value less than 0.05 was considered significant. Pearson linear regression was used to determine the degree of association between CTX-II levels, type II collagen expression, and Mankin scores. The linear regression coefficient, *R*^2^, is presented.

## 3. Results

### 3.1. Effect of Resveratrol on Body Weight and Serum Lipid Levels

**Figure 1:**
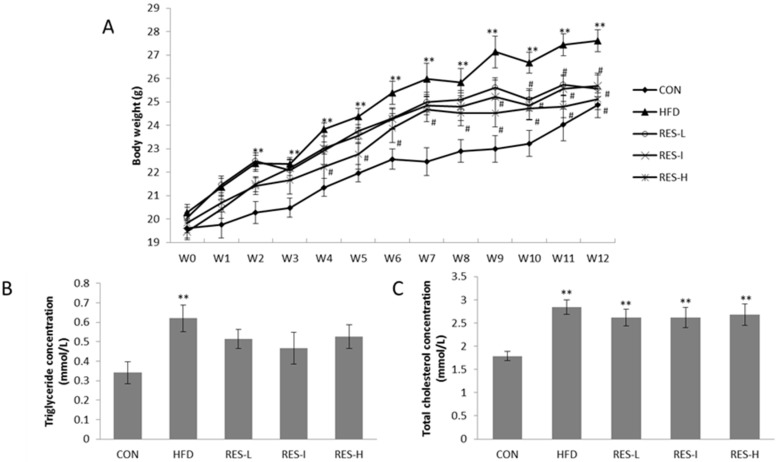
Effect of resveratrol on body weight and serum lipid levels. Male C57BL/6 mice were divided into five groups (*n* = 12 per group). Mice in the CON group received a standard diet containing 10% kcal from fat. Mice in the HFD group received a homemade HFD containing 58% kcal from fat. Mice in the RES-L, RES-I, and RES-L groups received a HFD with various doses of resveratrol by oral gavage for 12 weeks. (**A**) Body weights were measured weekly for 12 weeks; serum triglyceride levels (**B**); and serum total cholesterol levels (**C**) were measured after 12 weeks. All data are expressed as the mean ± SE. ANOVA was used to test for statistical significance. ** *p* < 0.01 *versus* the CON group; ^#^ *p* < 0.05 *versus* the HFD group.

Mice in the HFD group exhibited a significant increase in body weight between two weeks and 12 weeks after the start of the HFD diet compared with the CON mice (*p* < 0.01; Figure 1A). For the mice that received a HFD in combination with various doses of resveratrol, significantly higher body weights were observed at week two compared with the CON mice (*p* < 0.05; Figure 1A), yet these mice were only slightly heavier than the CON mice after 12 weeks (*p* > 0.05; Figure 1A). Conversely, mice in the RES-H, RES-I, and RES-L groups exhibited a significant reduction in body weight at weeks four, nine, and 10, respectively, compared with the HFD group (*p* < 0.05; Figure 1A). However, none of these differences between the three resveratrol-treated groups were significant. Moreover, resveratrol treatment had no influence on the amount of food intake (3.1 ± 0.5 g/day in the CON group *versus* 3.0 ± 0.2 g/day in the HFD group *versus* 3.0 ± 0.3 g/day in the RES-L group *versus* 3.1 ± 0.2 g/day in the RES-I group *versus* 3.2 ± 0.7 g/day in the RES-H group, *p* > 0.05).

Serum triglyceride and cholesterol concentrations were higher in the HFD group (*p* < 0.05), while serum triglyceride concentrations decreased by 17% in the RES-L group, 25% in the RES-I group, and 15% in the RES-H group, compared with the HFD group. However, none of these reductions were statistically significant (Figure 1B,C).

### 3.2. Histological Assessment of Knee OA in Resveratrol-Treated Mice

**Figure 2:**
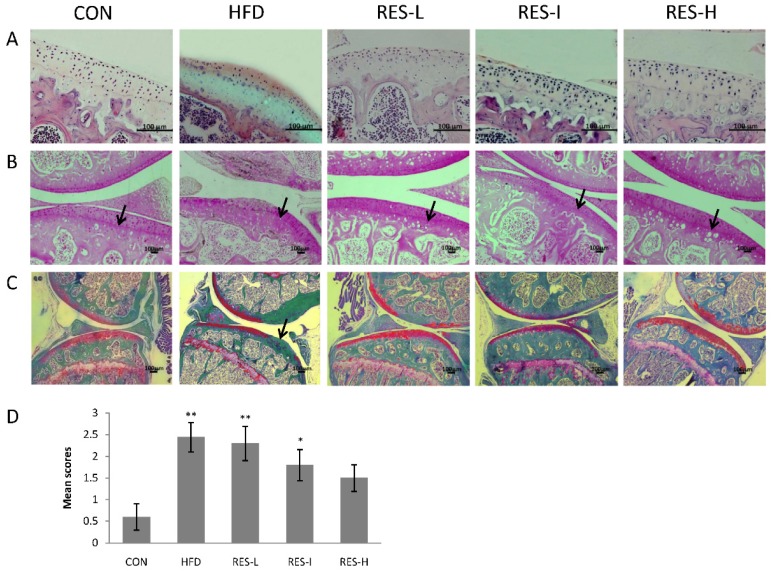
Histological analysis of knee articular cartilage. Representative sections from knee joints that were collected after 12 weeks and stained with: (**A**) H & E (original magnification, 200×); (**B**) Safranin O (original magnification, 100×); and (**C**) Safranin O/Fast Green (original magnification, 40×). Scale bar = 100 μm; (**D**) Quantification of modified Mankin scores. Data are expressed as the mean scores ± SE for 10 mice per group. Black arrows indicate the tide lines or articular cartilage lesion areas. ANOVA was used to test for statistical significance. * *p* < 0.05, ** *p* < 0.01 *versus* the CON group.

H & E, Safranin O, and Safranin O/Fast Green staining at 12 weeks revealed that the arrangement of cells in the knee joints of the HFD group was disordered and the matrix exhibited irregular staining. In addition, typical characteristics of OA were observed in the HFD group, including loss of tide lines, a reduction in cartilage thickness, and the presence of articular cartilage lesions in the medial tibial plateaus. In contrast, resveratrol treatment resulted in some improvement in matrix arrangement, tide line maintenance, or cartilage lesion inhibition (Figure 2A–C).

The severity of OA in each group was further evaluated using a Mankin scoring system. The Mankin score for the HFD group was markedly higher compared with the CON group (*p* < 0.01; Figure 2D). The scores for the resveratrol treatment groups exhibited varying degree of improvement compared to the HFD group (e.g., RES-L, 4%; RES-I, 25%; RES-H, 37%; *p* = 0.07).

### 3.3. Effect of Resveratrol on the Degradation of Type II Collagen in Knee Articular Cartilage

**Figure 3:**
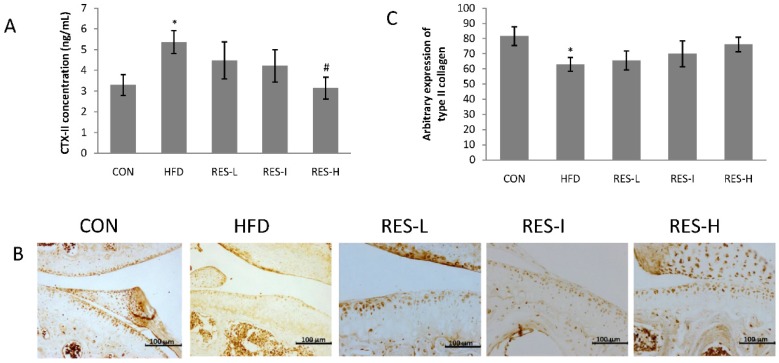
Effect of resveratrol on the degradation of type II collagen in knee articular cartilage. (**A**) Serum CTX-II levels were measured after 12 weeks; (**B**) Representative sections of knee joints that were stained for type II collagen (original magnification, 200×). Scale bar = 100 μm; (**C**) Histomorphometric measurements of type II collagen expression. Positive cells were defined based on type II collagen immunostaining. The whole cartilage areas were selected, quantified, and averaged. All data are expressed as the mean ± SE for 10 mice per group. ANOVA was used to test for statistical significance. * *p* < 0.05 *versus* the CON group and ^#^ *p* < 0.05 *versus* the HFD group.

CTX-II, a degradation product of type II collagen, may reflect the degree of articular cartilage damage that has occurred in the early stages of OA [27,28]. Therefore, serum levels of CTX-II were assayed. A significant increase in CTX-II serum levels were observed in the HFD group compared with the CON group (*p* < 0.05; Figure 3A). Conversely, mice in the RES-H group displayed significantly reduced levels of CTX-II compared with the HFD group (*p* < 0.05).

Based on the immunohistochemical analyses that were performed, lower levels of type II collagen were detected in the HFD group compared with the CON group (*p* < 0.05; Figure 3B,C). Moreover, the resveratrol treatment groups exhibited modest effects on the restoration of type II collagen expression compared with the HFD group (RES-L, 4%; RES-I, 11%; RES-H, 21%; Figure 3B,C).

### 3.4. Effect of Resveratrol on Chondrocyte Apoptosis

**Figure 4:**
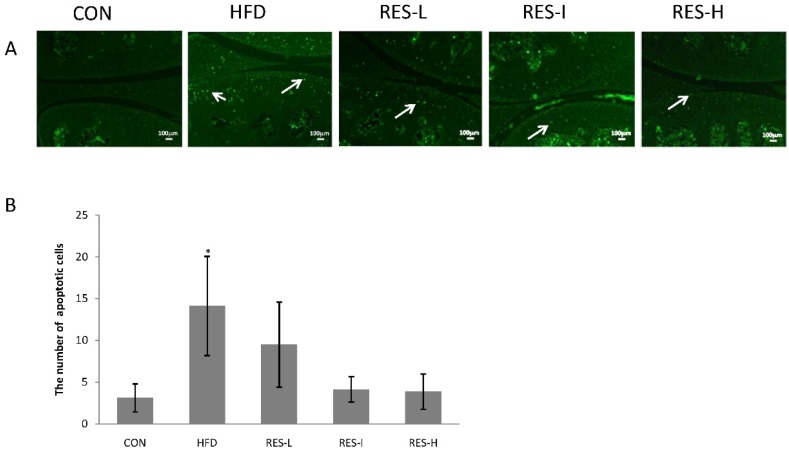
Effect of resveratrol on chondrocyte apoptosis. (**A**) Representative sections of articular cartilage were stained for TUNEL to analyze apoptosis (shown in green; original magnification, 100×). White arrows indicate the TUNEL-positive cells. Scale bar = 100 μm; (**B**) The number of TUNEL-positive cells (indicated with arrows) was counted. Data represent the mean ± SE of eight mice per group. ANOVA was used to test for statistical significance. * *p* < 0.05 *versus* the CON group.

TUNEL-positive cells were found to be abundantly expressed in the superficial layer of the articular cartilage in the HFD group, while the resveratrol-treated mice had fewer TUNEL-positive cells (Figure 4A). Quantification of the TUNEL stainings performed revealed that the number of positive cells was significantly higher in the HFD group compared with the CON group, while the number of TUNEL-positive cells was lower in the resveratrol-treated mice, especially in the RES-I and RES-H groups (Figure 4B).

### 3.5. Associations between Mankin Scores, Serum CTX-II Levels, and Type II Collagen Expression

**Figure 5:**
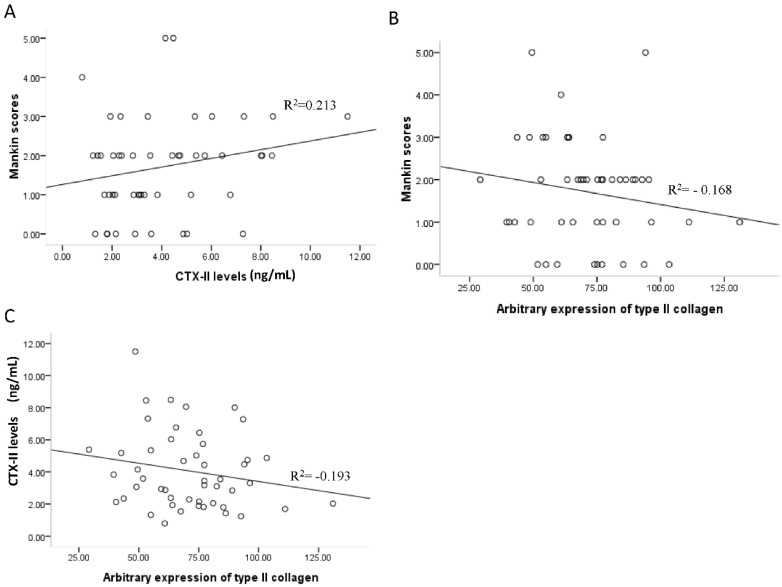
Correlation between Mankin scores, type II collagen expression, and serum CTX-II levels. Pearson’s correlation coefficient was calculated for each pair of parameters: (**A**) serum CTX-II levels and modified Mankin scores, *R*^2^ = 0.213; (**B**) type II collagen expression and modified Mankin scores, *R*^2^ = −0.168; and (**C**) type II collagen expression and serum CTX-II levels, *R*^2^ = −0.193. Each point on the graph represented a single mouse.

To date, no measurable diagnostic markers for OA have been established. Therefore, in order to explore a potential method for assessing OA in our animal model, we analyzed the association between Mankin scores, serum CTX-II levels, and type II collagen expression by regression analysis. When Pearson correlation coefficients were calculated, there was no correlation observed between Mankin scores, CTX-II levels, and type II collagen expression (shown in Figure 5, *p* > 0.05).

## 4. Discussion

The principal findings of the present study were: (i) a HFD administered to C57BL/6 mice for 12 weeks increased the incidence of knee OA; (ii) oral supplementation of resveratrol at doses of 22.5 mg/kg and 45 mg/kg partially inhibited or delayed the development of OA by decreasing body weight, reducing degradation of type II collagen, and suppressing chondrocyte apoptosis.

HFD-induced susceptibility to OA depends on mouse strain and gender [29]. Several groups have developed models of knee OA by feeding male C57BL/6 mice a diet containing a high fat content [18,30]. In the present study, mice in the HFD group more frequently developed OA compared with the control mice, thereby demonstrating successful recapitulation of obesity-related OA. Moreover, while a HFD was administered in the present study from the age of seven weeks to 19 weeks, previous studies have administered a HFD to mice aged six- or 12-weeks old for the duration of their lives [30]. Different proportions of fat in a diet may also affect the onset of OA, with a higher fat content associated with accelerated occurrence and progression of OA. In the present study, mice in the HFD group that developed OA exhibited significantly greater body weights compared with the control mice that received a standard diet, thereby supporting a link between body weight and OA. However, the Mankin scores revealed that three mice in the control group developed OA even though their body weights were similar to the mice that did not develop OA, which indicates that weight, alone, is not a causal factor underlying the etiology of HFD-induced OA [19].

The biological effects of resveratrol have been well documented in the literature. The therapeutic potential of resveratrol is reflected by its anti-oxidant, anti-apoptotic, and anti-inflammatory capacities, as well as its neuroprotective and cardioprotective roles [4,5,6,31,32]. Therefore, resveratrol may provide a safe alternative to current pharmacological therapies for OA. The potential beneficial effects of resveratrol in OA models were first reported in a rabbit model established by Elmali and colleagues [33] where degeneration in the knee was induced by unilateral knee ligament transaction. Following the administration of intra-articular injections of resveratrol, a significant reduction in the extent of cartilage tissue destruction and proteoglycan loss were observed [33]. Similarly, intra-articular injections of resveratrol in another study prevented OA progression as evidenced by retained expression of collagen type II and reduced levels of inducible nitric oxide synthase and matrix metalloproteinase-13 [8]. Taken together, these findings suggest that intra-articular injections of resveratrol may protect cartilage against the development of experimentally-induced inflammatory and degenerative arthritis. However, these studies did not indicate whether oral administration of resveratrol would also provide OA-protective effects.

In the present study, we administrated resveratrol to mice by oral gavage. This method is associated with minimal trauma and high compliance. Body weights in the resveratrol-treated groups were all significantly decreased, suggesting that resveratrol may exert anti-OA effects in obese mice partly by reducing biomechanical overloading and inflammatory factors.

In addition to body weight, serum CTX-II levels and type II collagen expression were also assessed to determine the effect of resveratrol on OA. Serum levels of CTX-II have been shown to positively correlate with cartilage degeneration [27,28], and have also been useful as a diagnostic and prognostic biomarker [34] for evaluating treatment efficacy in knee OA [35]. In the present study, high doses of resveratrol (45 mg/kg) significantly decreased serum levels of CTX-II, thereby indicating that resveratrol relieved cartilage degradation in mice that received a HFD. Type II collagen is a dominant component of the extracellular matrix of cartilage and, upon degradation by catabolic enzymes, CTX-II is released and gains access to blood, urine, and bodily fluids [36]. Resveratrol treatment, especially at high doses, prevented the degradation of type II collagen that was induced by the HFD, and this effect partly contributed to the delay in OA progression. Furthermore, when a possible correlation between CTX-II, type II collagen, and Mankin scores were examined, no correlation was observed. Thus, dynamic, reliable, and quantitative tests to detect early damage and to measure the progress of treatments targeted against joint destruction remain to be identified.

It is reported that an abundance of apoptotic cells can be found within the chondro-osteophyte at the periphery of OA joints, even in the early stages of disease [37]. Moreover, increases in chondrocyte apoptosis have been anatomically linked to proteoglycan depletion [38]. *In vitro*, resveratrol has been shown to inhibit chondrocyte apoptosis induced by IL-1β-stimulated inflammation in human articular chondrocytes [4,39,40]. A recent study showed that oral resveratrol treatment in leptin deficient ob/ob mice reduced the area of calcified cartilage in the proximal tibial growth plate [41]. This result may be due to the decreasing effects of resveratrol on angiogenic factors, which are in charge of facilitating calcification of growth plate cartilage [42]. Based on these observations, we investigated the effect of resveratrol on chondrocyte apoptosis in the mice that received a HFD. Knee joints of the HFD group contained a large number of apoptotic chondrocytes, while the knee joints of the mice that received resveratrol had fewer apoptotic chondrocytes. These differences were not statistically significant due to the large differences observed within the same group. However, the average numbers of apoptotic chondrocytes in the RES-I group (*p* = 0.069) and the RES-H group (*p* = 0.062) were markedly lower compared with the HFD group. Taken together, these results indicate that resveratrol mediates at least a modest anti-apoptotic effect on obesity-related OA. To our knowledge, our findings are the first to demonstrate that oral resveratrol exerts anti-OA effects by decreasing body weight, by retaining type II collagen, and by reducing chondrocyte apoptosis in mice fed a HFD. Therefore, the potential for resveratrol to serve as a preventive or therapeutic strategy for obesity-related OA should be further investigated, particularly the administration of oral resveratrol. Regarding the latter, a HFD should be administered over a longer period of time, and appropriate dosing and administration schedule should be optimized.

## 5. Conclusions

In conclusion, our findings demonstrated that a HFD administered to C57BL/6 mice increased the incidence of knee OA, and oral resveratrol partially inhibited the development of OA by decreasing body weight, reducing degradation of type II collagen, and suppressing chondrocyte apoptosis. Thus, the administration of oral resveratrol appears to exert anti-OA effects in a mouse model of HFD-induced OA, thereby highlighting the potential preventive or therapeutic value of resveratrol for obesity-associated OA.
